# Supplementary material for: Extensive Copy Number Variation in Fermentation-Related Genes Among Saccharomyces cerevisiae Wine Strains
Source: G3 (Bethesda). 2017 Mar 13;7(5):1475–85. doi: 10.1534/g3.117.040105 (PMC5427499; doi:10.1534/g3.117.040105)
Supplement: Supplementary file 2 [file 1475FileS2.docx]

**Supplementary Tables and Figures to**

**Extensive Copy Number Variation in Fermentation-Related Genes among *Saccharomyces cerevisiae* Wine Strains**

Jacob Steenwyk and Antonis Rokas^*^

Department of Biological Sciences, Vanderbilt University, Nashville, TN 37235, USA

*Correspondence: [antonis.rokas@vanderbilt.edu](mailto:antonis.rokas@vanderbilt.edu)

|  | **Average** | **Median** | **Max** | **Min** |
| --- | --- | --- | --- | --- |
| **CNVR Size (bp)** | 5345.53  (± 8563.04) | 500 | 416,500 | 500 |
| **CNVRs** | 97.78  (± 9.46) | 86 | 126 | 78 |
| **CNVRome** | 0.04299  (± 0.01) | 0.04059 | 0.09802 | 0.03056 |

**Table S1. Summary statistics of CN variable regions (CNVRs) in wine yeast strains.**

| **Subject Name(s)** | **Subject Seq ID** | **E-value** | **Bitscore** | **Num. of Strains** |
| --- | --- | --- | --- | --- |
| Saccharomyces cerevisiae YJM1381 | gi\|767173083\|gb\|AJS47322.1\| | 0 | 6528 | 1 |
| Saccharomyces cerevisiae x Saccharomyces kudriavzevii VIN7 | gi\|365761738\|gb\|EHN03375.1\| | 0 | 4263 | 1 |
| Saccharomyces cerevisiae YJM195;Saccharomyces cerevisiae YJM1248;Saccharomyces cerevisiae YJM1439 | gi\|767223679\|gb\|AJT02268.1\| | 0 | 4149 | 1 |
| Saccharomyces cerevisiae YJM1418 | gi\|768799386\|gb\|AJV24208.1\| | 0 | 3826 | 1 |
| Saccharomyces cerevisiae YJM1400;Saccharomyces cerevisiae YJM1479 | gi\|766481881\|gb\|AJS05941.1\| | 0 | 3735 | 1 |
| Saccharomyces cerevisiae S288c | gi\|1708192\|sp\|P32874.2\|HFA1_YEAST | 0 | 3660 | 1 |
| Saccharomyces cerevisiae YJM1389;Saccharomyces cerevisiae YJM1460;Saccharomyces cerevisiae YJM1592 | gi\|768798675\|gb\|AJV23504.1\| | 0 | 3545 | 1 |
| Saccharomyces cerevisiae YJM627 | gi\|767191293\|gb\|AJS69960.1\| | 0 | 2987 | 1 |
| Saccharomyces cerevisiae;Saccharomyces cerevisiae YJM1273;Saccharomyces cerevisiae YJM1338;Saccharomyces cerevisiae YJM1386 and others | gi\|767240124\|gb\|AJT18667.1\| | 0 | 2929 | 1 |
| Saccharomyces cerevisiae EC1118 | gi\|259149880\|emb\|CAY86684.1\| | 0 | 2917 | 7 |
| Saccharomyces cerevisiae YJM1252 | gi\|768526763\|gb\|AJU39807.1\| | 0 | 2750 | 1 |
| Saccharomyces cerevisiae;Saccharomyces boulardii;Saccharomyces cerevisiae EC1118;Saccharomyces cerevisiae AWRI796 and others | gi\|259149028\|emb\|CAY82271.1\| | 0 | 2536 | 8 |
| Saccharomyces cerevisiae x Saccharomyces kudriavzevii VIN7 | gi\|365758302\|gb\|EHN00152.1\| | 0 | 2308 | 1 |
| Saccharomyces cerevisiae YJM1307 | gi\|767203839\|gb\|AJS82476.1\| | 0 | 2222 | 1 |
| Saccharomyces cerevisiae YJM193;Saccharomyces cerevisiae YJM271 | gi\|768459294\|gb\|AJT71561.1\| | 0 | 2177 | 1 |
| Saccharomyces cerevisiae;Saccharomyces cerevisiae S288c;Saccharomyces cerevisiae YJM428;Saccharomyces cerevisiae YJM1083 and others | gi\|330443503\|ref\|NP_010181.2\| | 0 | 2166 | 1 |
| Saccharomyces cerevisiae YJM1386 | gi\|767209693\|gb\|AJS88316.1\| | 0 | 2111 | 1 |
| Saccharomyces cerevisiae YJM1418 | gi\|768539331\|gb\|AJU52326.1\| | 0 | 2096 | 1 |
| Saccharomyces cerevisiae x Saccharomyces kudriavzevii VIN7 | gi\|365758942\|gb\|EHN00762.1\| | 0 | 2094 | 1 |
| Saccharomyces cerevisiae x Saccharomyces kudriavzevii VIN7 | gi\|365760689\|gb\|EHN02393.1\| | 0 | 2077 | 1 |

**Table S2. Top 20 loci absent in S288c.** The 20 loci with the highest bitscores that mapped to various *Saccharomyces cerevisiae* strains are presented here. Columns are arranged by subject name (what the scaffolds’ best blast match was), subject sequence ID, e-value, bitscore and the number of the 20 strains examined that had the locus absent in S288c.


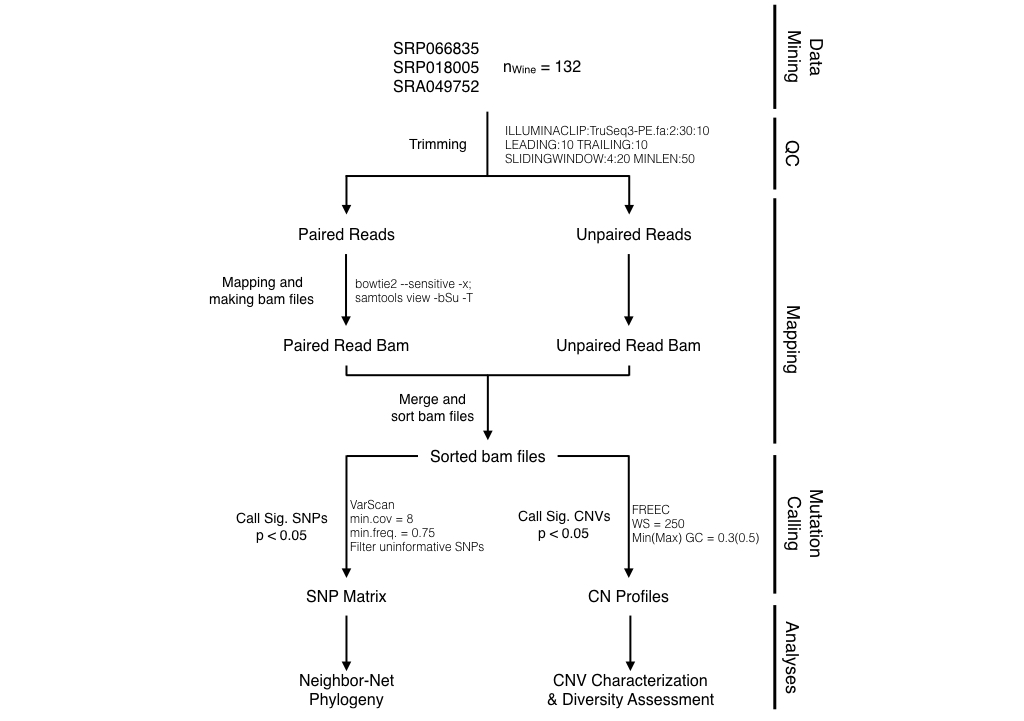


**Figure S1. Workflow of analyses performed in this study.** Whole genome Illumina paired-end sequence reads from 132 wine yeast strains were obtained from three different studies. The reads of all strains underwent the same trimming and mapping processes. Trimming produced paired and unpaired sequence reads that were mapped to *S. cerevisiae* S288C separately. Resulting bam files were merged to capture as much read depth information as possible. Statistically significant (*p* < 0.05) single nucleotide polymorphisms (SNPs) were identified with VarScan and utilized in Neighbor-Net phylogenetic analysis. Statistically significant CN variants (*p* < 0.05) relative to the S288C genome were identified and quantified using the read depth based approach implemented by Control-FreeC. The resulting CNVs were used in downstream characterization and diversity assessment analysis.


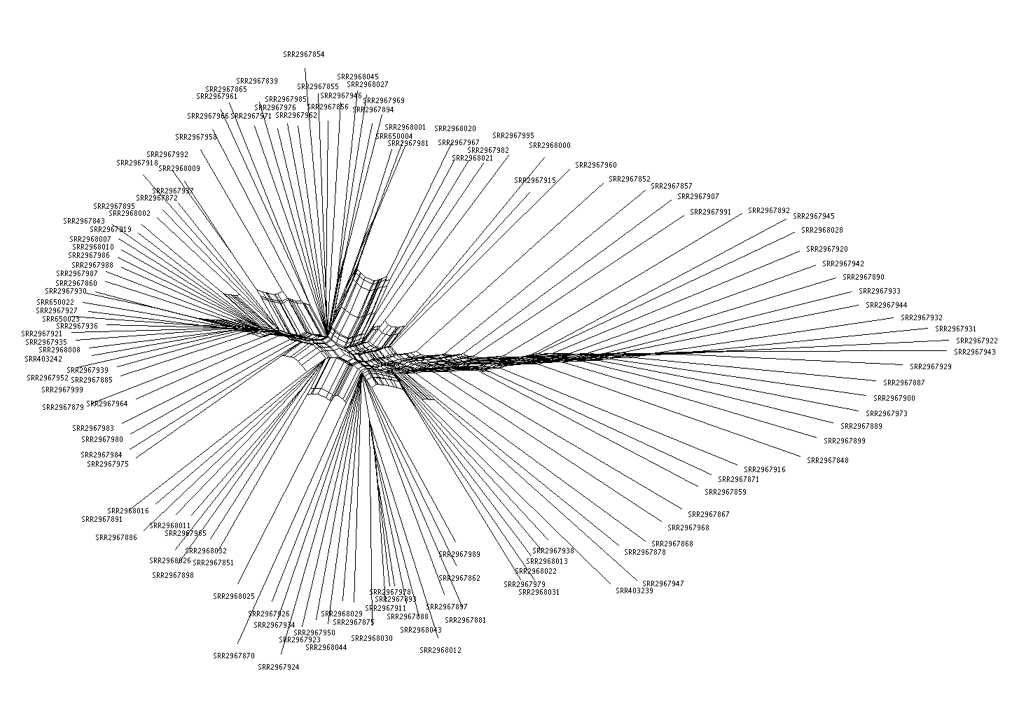


**Figure S2. Neighbor-Net phylogeny of wine yeast strains.** Neighbor-Net phylogenetic network analyses from an informative set of 43,370 SNPs. The proposed relationships among strains appear similar to those in the maximum-likelihood phylogeny reported by Borneman *et al.* 2016. As expected, wine yeast strains exhibit low levels of SNP genetic diversity.


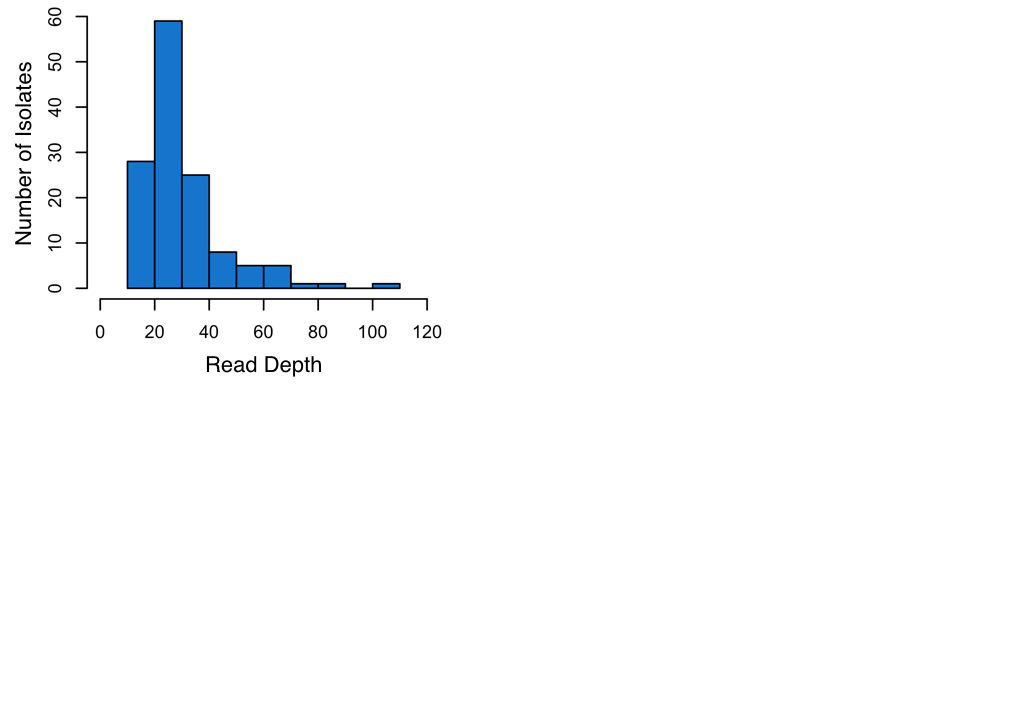


**Figure S3. Distribution of sequence read depths of wine yeast strains.** As part of the quality assessment of the genome data of wine yeast strains, sequencing read depth was quantified. The basic statistics of read depth were: minimum: 13.0, maximum: 104.5, median: 25.8, and average: 30.1.


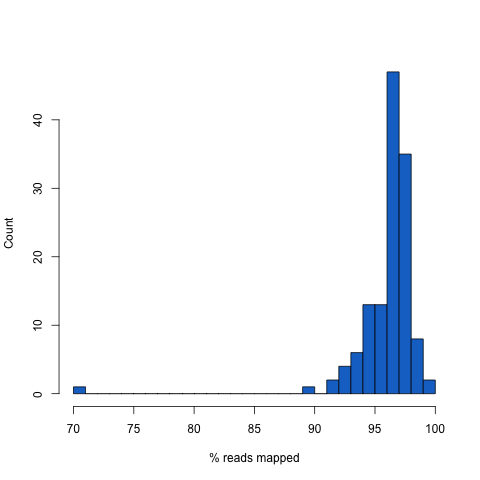


**Figure S4. Distribution of reads mapped to S288C.** To identify candidate strains with the most unrepresented loci, the percent of reads mapped per strain was calculated and the twenty with the least mapped reads were further analyzed. The basic statistics of percent reads mapped were: minimum: 70.5%, maximum: 99%, median: 96.6%, and average: 96%.


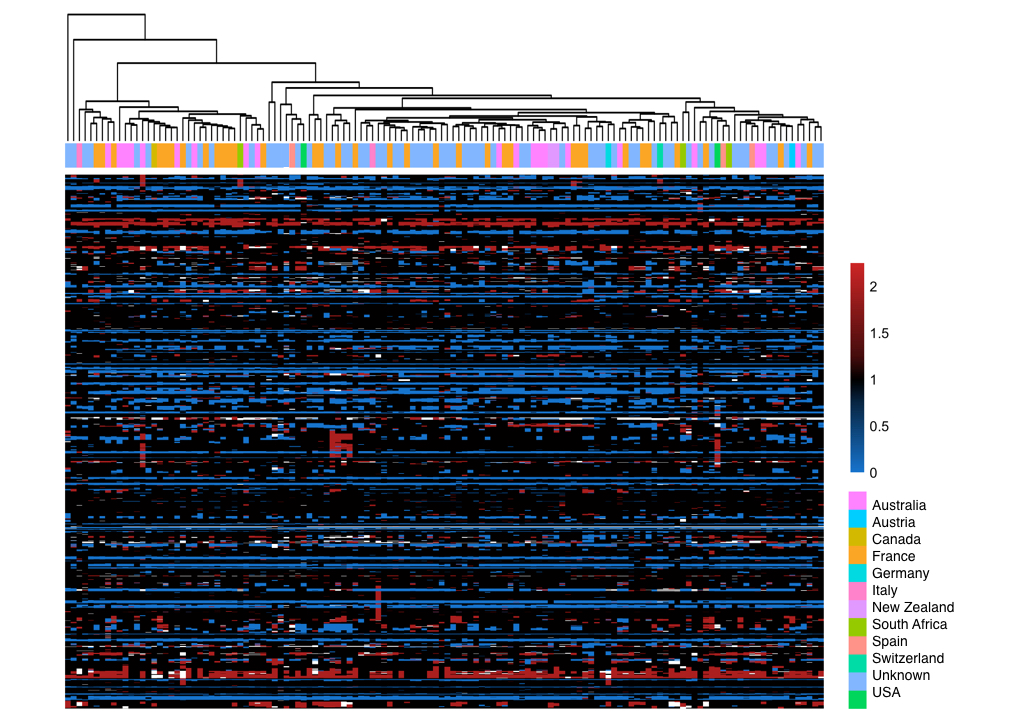


**Figure S5. Whole genome CN profiles of 132 wine yeast strains.** Whole genome CN profiles were generated for each strain, as described in the Methods section. 2,820 CN variable loci that overlap with 2,061 genes are shown here with individual strains as columns and loci as rows. Blue heat map colors indicate deletions, black indicate absence of CN variation, red indicate a single duplication, and white indicate two or more duplications. Geographic origin of strains is provided underneath the dendrogram.


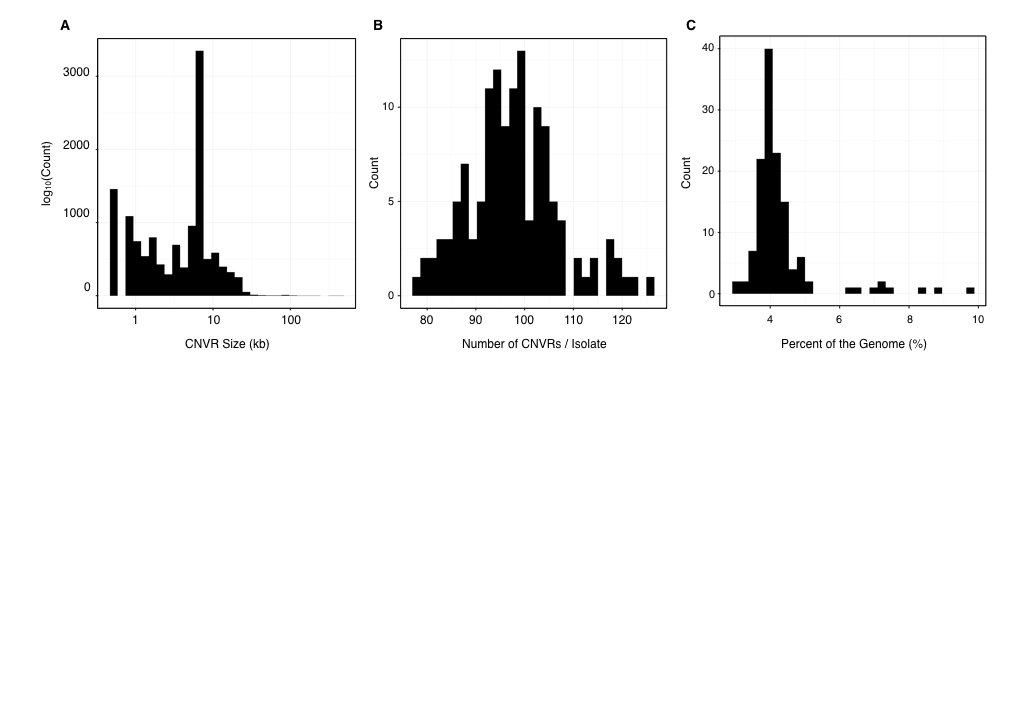


**Figure S6. Population distributions of size and number of CN variable regions as well as of the fraction of genome they affect.** (A) The log_10_(count) of CN variable regions (CNVRs) is plotted against CNVR size (in kb); most CNVRs are 10 kb or less in length. (B) The distribution of the number of CNVRs / strain; most strains have ~100 CNVRs. (C) The distribution of the fraction of the genome of each strain that is affected by CNVRs; most strains have ~3-5% of their genome affected by CN variants.


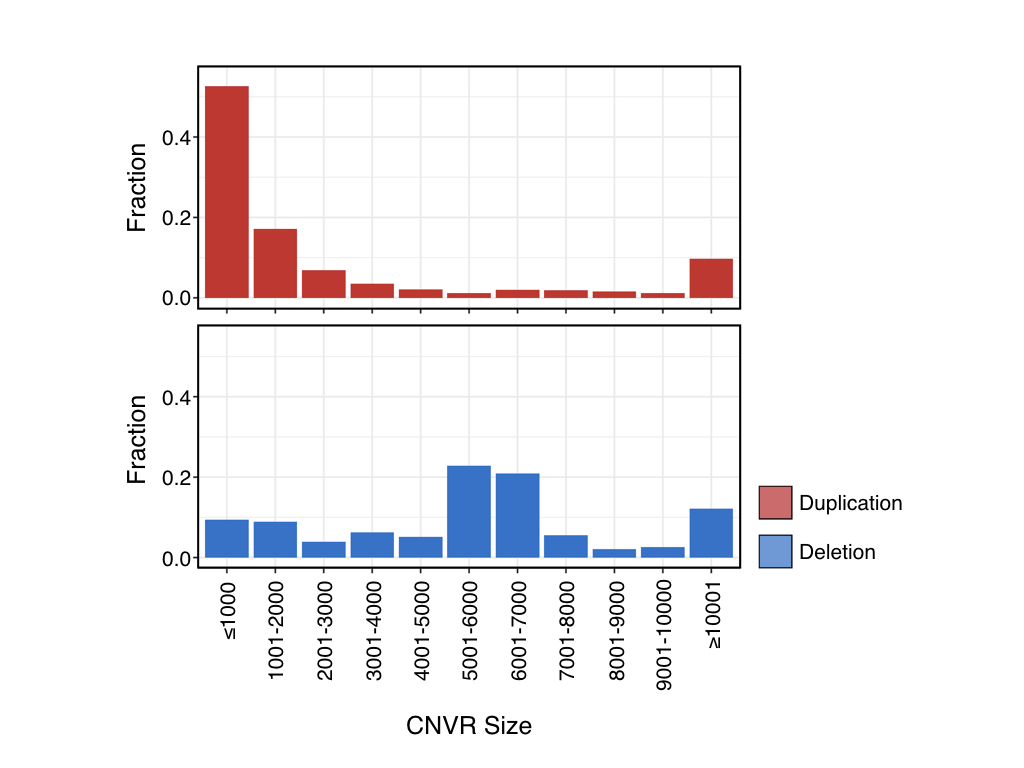


**Figure S7. Fraction of CNVRs for a given size range for duplications and deletions.** (A) The majority of duplications is less than 1 kb in size and (B) the majority of deletions is between 5-7 kb. These deletions were flanked by Ty transposable elements.


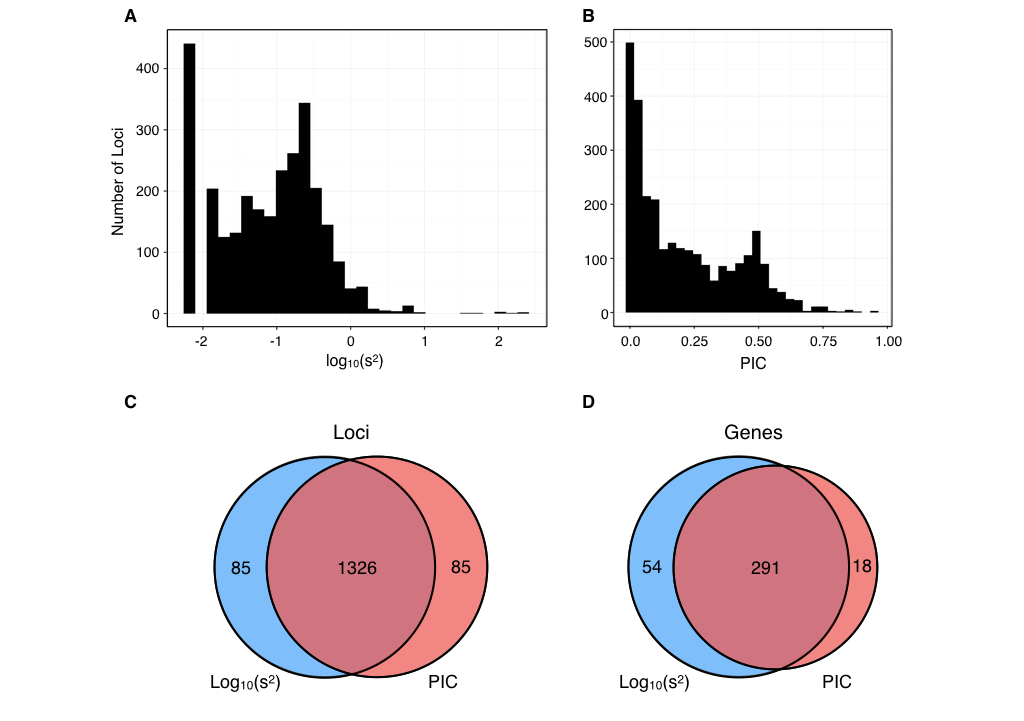


**Figure S8. Distribution of log_10_(s^2^) and PIC values and the number of shared loci and genes.** For each CN variable locus, log_10_(s^2^) (panel A) and PIC (panel B) was calculated. High log_10_(s^2^) or PIC values are considered to be in the top 50th percentile of log_10_(s^2^) or PIC values. Loci harboring high log_10_(s^2^) and PIC values were merged and used downstream analysis. (C and D) Both methods of CN diversity identified many of the same loci and genes, however, each method was also able to identify a few additional loci and genes.


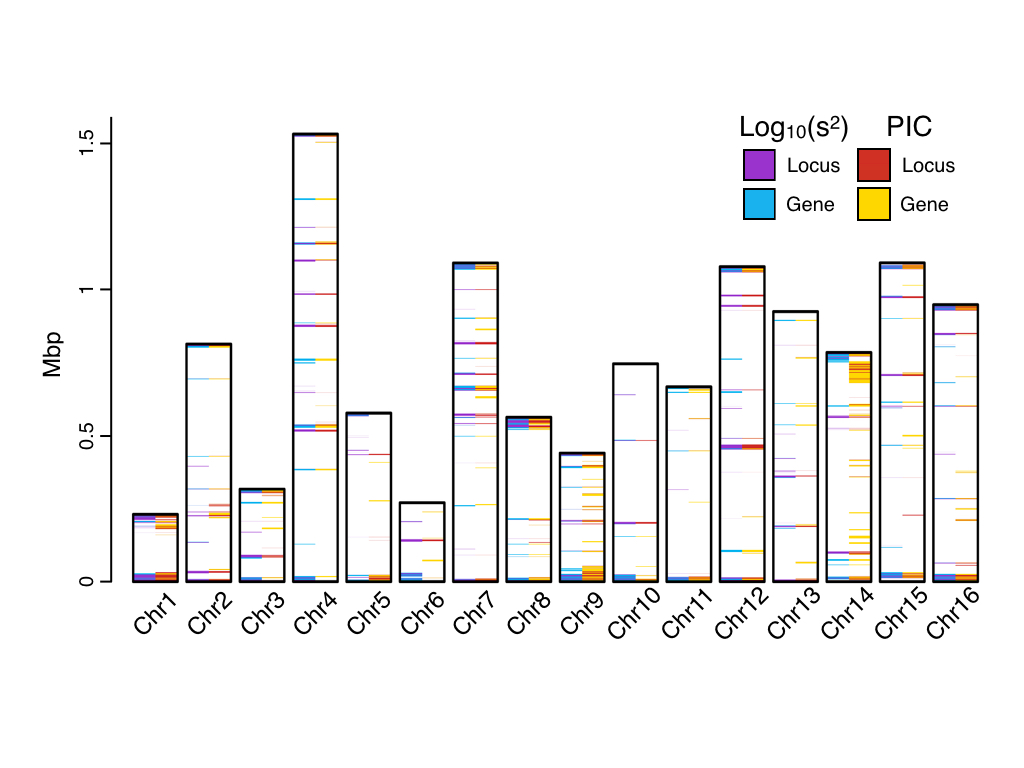


**Figure S9. Genomic distribution of CN diverse loci and genes.** CN diverse loci and genes identified via the log_10_(s^2^) approach are depicted in purple and blue, respectively, across the 16 nuclear chromosomes of yeast. CN diverse loci and genes identified by the PIC approach are shown in red and yellow, respectively.


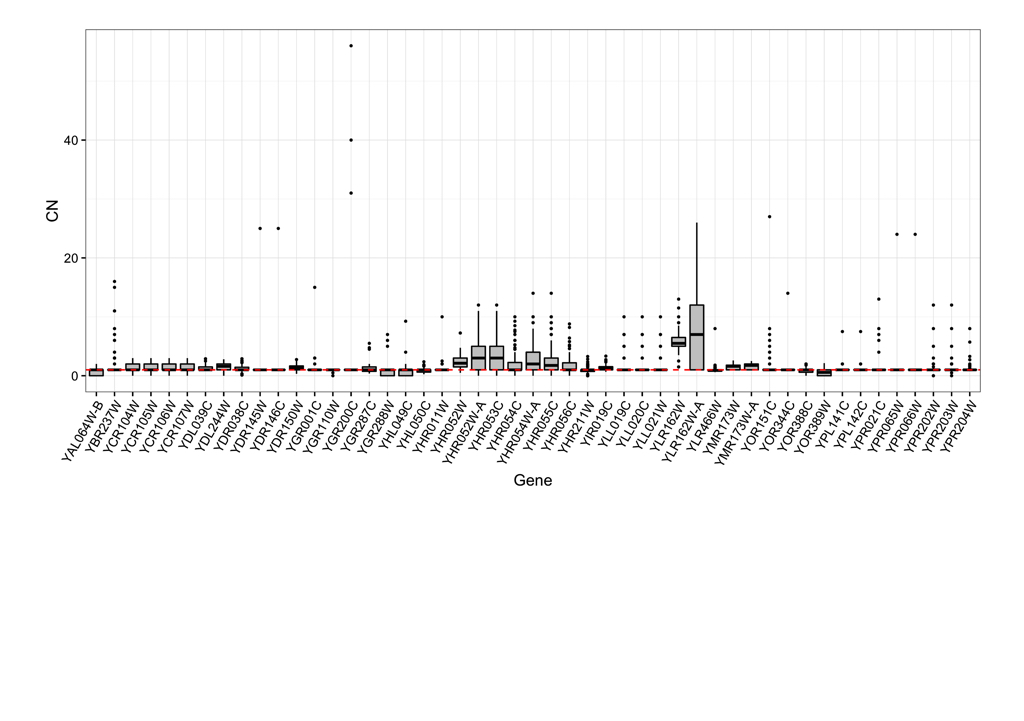


**Figure S10. CN of genes with high CN diversity values.** 57 CN diverse genes were identified as described in the Methods section. The CN profiles of all 57 genes are shown here excluding those found as elements of the ribosomal DNA locus for scaling purposes. CN distributions are shown along the x-axis and can range from 0 (deletion) to greater than 50. The dashed red line represents a CN of 1.


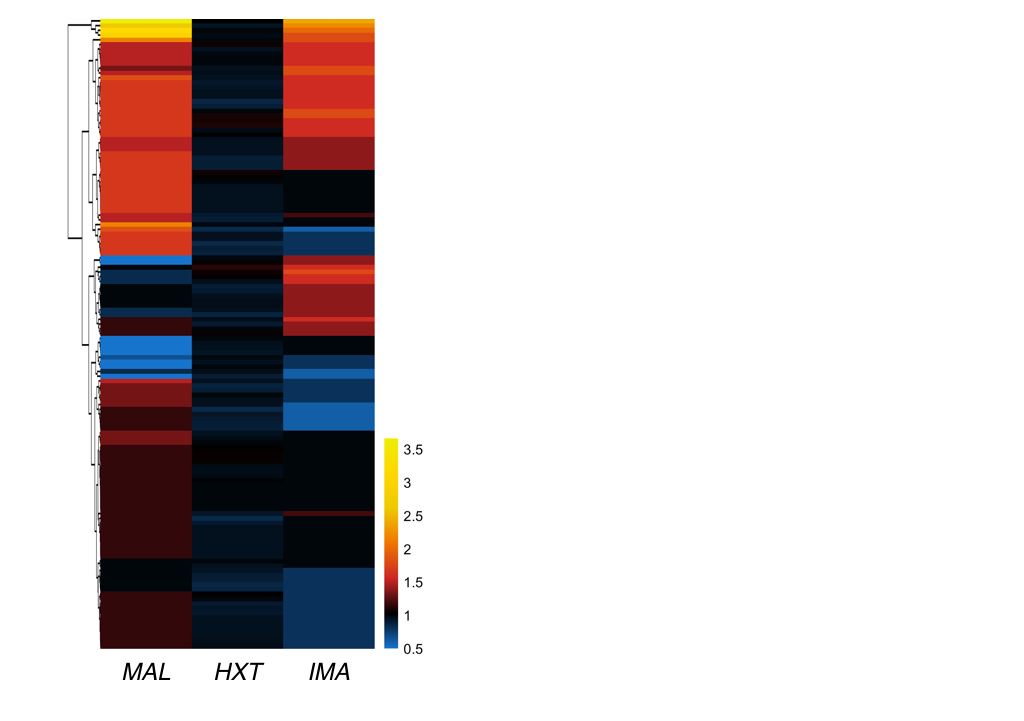


**Figure S11. CN diversity of three gene families involved in fermentation.** To calculate the CN of each gene family in each strain, we first calculated the sum of the CN of each member of the *MAL, HXT,* and *IMA* gene families in each strain and then divided this sum by the number of genes present in each gene family. For example, there are 19 *HXT* genes so the CN of *HXT* genes was summed for each strain and then divided by 19. If all genes are present in one copy in a given strain (the different strains are shown as different rows), then the corresponding row will be black. If the average number of genes in a gene family is less than one, then the row will be colored blue (indicating that the gene family is contracted in this strain), and if the average number of genes is more than one then the row will be colored red or yellow (indicating that the gene family is expanded in this strain). Note the mosaic patterns of expansions and contractions in the *MAL* and *IMA* gene families and the minor contraction pattern of the *HXT* family.


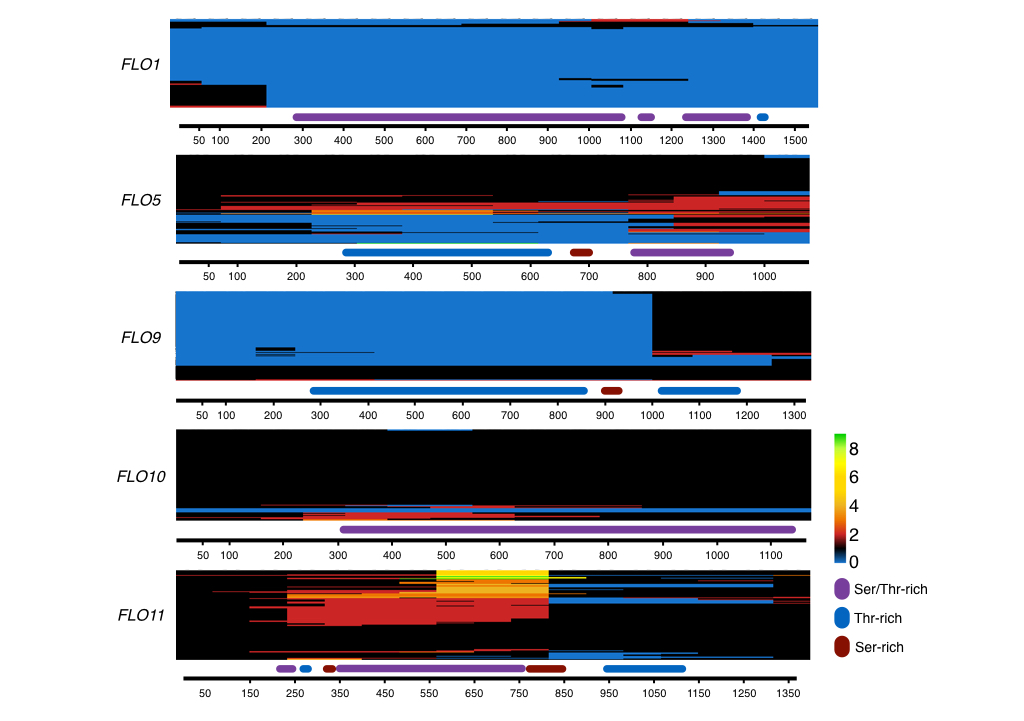


**Figure S12. CN Profiles of members of the *FLO* gene family.** The CN variation of the Serine / Threonine-rich regions of members of the *FLO* gene family were investigated using a 250 bp sliding window approach where rows represent individuals and columns represent 250 bp windows. Some *FLO* genes have similar CN profiles between strains (e.g., *FLO1, FLO10*) while others have more variable CN profiles (e.g., *FLO5, FLO11*). *FLO11* appears to have significant CN variation in the Serine / Threonine-rich region thought to control flocculation. Black color indicates no CN variation, blue indicates a deletion, and red-green colors represent duplications.


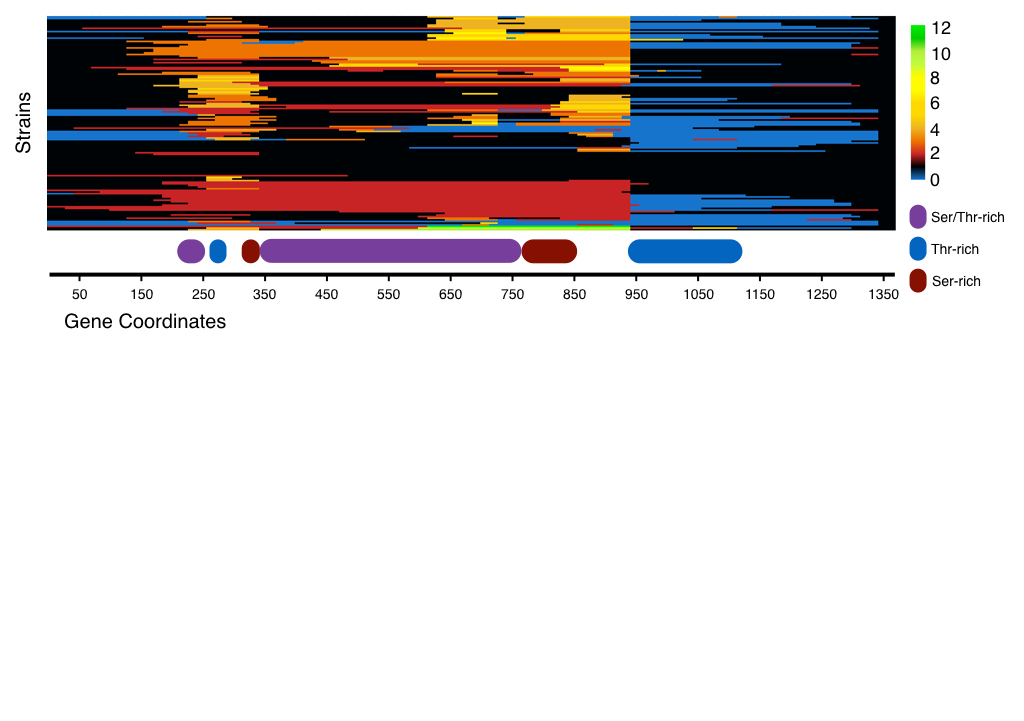


**Figure S13. CN variation within the *FLO11* gene.** To capture CN variation within the tandemly repeated regions of the *FLO11* gene, we used a 25 bp sliding window approach. CN variation in tandem repeat mutations can range from deletions to significant duplications (12 repeats) and is primarily observed in the Serine and/or Threonine-rich regions. Deletions are represented by blue, absence of CN variation by black, and duplications by red-green colors. Rows represent strains and columns genomic windows.


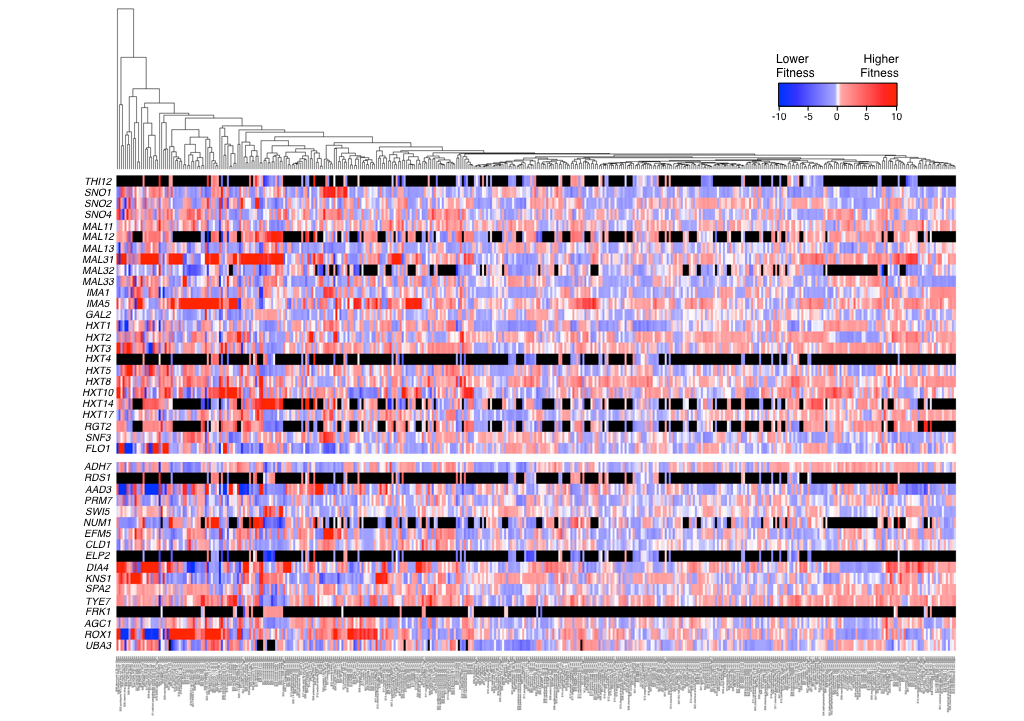


**Figure S14. The growth fitness effects of deletions of highly diverse CN genes across diverse environments.** Growth fitness gain or loss across 418 conditions compared to WT (S288C) is represented by red or blue, respectively, from the gene deletion study conducted by Hillenmeyer *et al.* 2010. The genes presented on the Y axis are the 42 genes of interest for which data were available and are split into two portions: the top half represents genes of interest identified by GO enrichment analysis (Figure 4) and the bottom half represents genes with high PIC or log_10_(s^2^) values. Cells colored blue indicate lower growth fitness relative to the wild-type strain, red cells indicate higher growth fitness, and black cells indicate the lack of growth fitness data. Deletion of any of the 42 genes we examined increased growth fitness in at least one condition and decreased growth fitness in at least another condition.


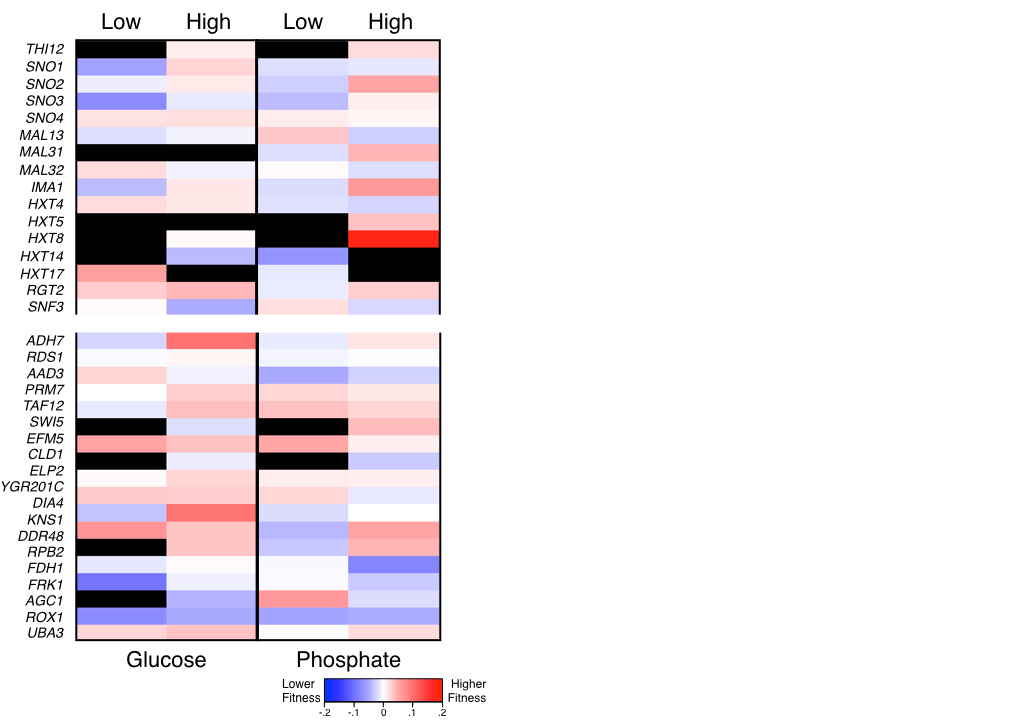


**Figure S15. The growth fitness effects of duplications of highly diverse CN genes in two fermentation-related environments.** The growth fitness effects of duplications was examined using low and high gene CN plasmids compared to WT (S288C) by Payen *et al.* 2016. The genes presented on the Y axis are the 35 genes of interest for which data were available and are split into two portions where top half are genes of interest identified by GO enrichment analysis (Figure 4) and the bottom half are genes with high PIC or log_10_(s^2^) values. Fitness decreases compared to wild-type are depicted in blue, fitness gains in red, and missing data in black. Presence of multiple copies of a gene can result in both fitness decreases and increases, depending on the given condition and the extent of plasmid CN.
